# God’s Decree (Poetry)

**Published:** 1881-06

**Authors:** 


### GOD'S DECREE.

It is ordained by God's decree
That all, however dear they be,
Must sever! Ah, sever!
Tho' nought there be that grieves the heart
So keenly as from friends to part,
And sever! Ah, sever.

A tender flower should'st thou receive,
And in a vase the token leave—
Remember! Remember!—
Ere morn the bud will open bright;
But wither with the shades of night!
Remember! Remember!

A cherished fond one, should there be,
Whom thou dost love most tenderly,—
Thine only!
Ah! ere a little time be flown
She'll die, and leave thee all alone—
So lonely! Ah, lonely!

Tho' nought there be that grieves the heart
So keenly as from friends to part,
And sever! Ah, sever!
Yet deem not thou my words in vain,
When those that love must part in twain,
But say, "We hope to meet again!"
To meet again!—Forever.

[*Translated from the German.*]

